# Precise diagnosis of acute mesenteric ischemia using indocyanine green imaging prevents small bowel resection: A case report

**DOI:** 10.1016/j.ijscr.2022.107463

**Published:** 2022-07-30

**Authors:** Kohei Furusawa, Masanori Yoshimitsu, Hiroyoshi Matsukawa, Kuniomi Oi, Keiji Yunoki, Akihisa Tamura

**Affiliations:** a Department of Surgery, Hiroshima City Hiroshima Citizens Hospital, 7-33, Moto-machi, Naka-ku, Hiroshima-shi, Hiroshima-ken 730-8518, Japan; b Department of Cardiology, Hiroshima City Hiroshima Citizens Hospital, 7-33, Moto-machi, Naka-ku, Hiroshima-shi, Hiroshima-ken 730-8518, Japan; c Department of Cardiovascular Surgery, Hiroshima City Hiroshima Citizens Hospital, 7-33, Moto-machi, Naka-ku, Hiroshima-shi, Hiroshima-ken 730-8518, Japan; d Department of Radiology, Hiroshima City Hiroshima Citizens Hospital, 7-33, Moto-machi, Naka-ku, Hiroshima-shi, Hiroshima-ken 730-8518, Japan

**Keywords:** Acute mesenteric ischaemia, Superior mesenteric artery thrombosis, Indocyanine green, Small-bowel resection, Case report

## Abstract

**Introduction:**

Acute mesenteric ischemia (AMI) is a rare life-threatening condition that causes intestinal necrosis. Prompt intervention is essential to mitigate high mortality. In this report, we describe a case of AMI where precise diagnosis using indocyanine green (ICG) imaging to confirm sufficient bowel perfusion and viability, helped in preventing intestinal resection.

**Presentation of case:**

A 91-year-old male was diagnosed with AMI associated with superior mesenteric artery thrombosis using computed tomography and underwent exploratory laparotomy. Under white light, there was no outward evidence of small-bowel necrosis. Hence, ICG was used to confirm adequate bowel perfusion and viability. The operation was terminated without resection of the small intestine. When anticoagulation therapy was initiated postoperatively, the thrombus subsided. Although the patient had no subsequent recurrence, he died of dysphagic pneumonia two months after the surgery.

**Discussion:**

Physicians often choose to perform trial laparotomy to diagnose intestinal ischemia due to AMI.

However, it was difficult to assess the viability of the entire intestinal tract using white light alone, and the introduction of ICG in the evaluation of intestinal perfusion will facilitate the identification and objective evaluation of the intestinal ischemic zone. There have been few reports on application of fluorescent-guided determination of the viable zone of the small intestine, which will help surgeons to make precise diagnosis.

**Conclusion:**

This case demonstrates ICG fluorescence imaging as a useful method for objectively assessing bowel viability.

## Highlights

• Laparotomy was performed in a patient with acute mesenteric ischemia.
• Indocyanine green was used since necrosis was not detected under white light.
• Precise diagnosis using Indocyanine green prevented small bowel resection.

## 1 Introduction

Acute mesenteric ischemia (AMI) is a rare and life-threatening disease that is characterized by sudden interruption of blood supply to a segment of the small intestine, leading to ischemia, cellular damage, and intestinal necrosis [1], [2], [4], [5]. Symptoms vary from patient to patient but include diffuse abdominal pain unrelated to physical symptoms, abdominal tenderness, nausea, vomiting, and diarrhea. The etiology of AMI includes advanced age, atherosclerosis, low cardiac output, arrhythmias, cardiovascular disease, intra-abdominal tumors, and increased incidence of cardiac disease, diffuse atherosclerosis, inflammatory bowel disease, and aging of the population [1], [5]. Diagnosis is based largely on clinical findings, results of computed tomography (CT) angiography, and visual inspection of bowel laparotomy or laparoscopy [2], [5]. Bowel resection is the only surgical option for necrotic bowel [3], [5].

Despite advancement in open and endovascular treatment options, it has an incredibly high mortality rate of 40 %–69 % [1], [2]. Unfortunately, mesenteric ischemia has no specific biomarkers. Patients usually present with non-specific abdominal pain, which can be caused by a large number of pathological conditions [2].

Prompt diagnosis and interventions are essential to mitigate high mortality [1], [2]. According to some studies, the mortality rate was 10.6 % if patients were operated on within 24 h after the onset of symptoms vs. 72.9 % if operated on after 24 h. Early detection is important, and the intestines with less advanced necrosis may be salvaged by revascularization, thereby increasing survival [2], [4]. In the current report, we describe a case of AMI diagnosed with laparoscopy with indocyanine green (ICG) fluorescence imaging to confirm sufficient bowel perfusion and viability. Our methodology prevented intestinal resection in the patient. This study will help in the precise diagnosis and better management of AMI.

The case is reported in accordance with the SCARE 2018 guidelines of reporting surgical cases [6].

## 2 Presentation of case

**Fig. 1:**
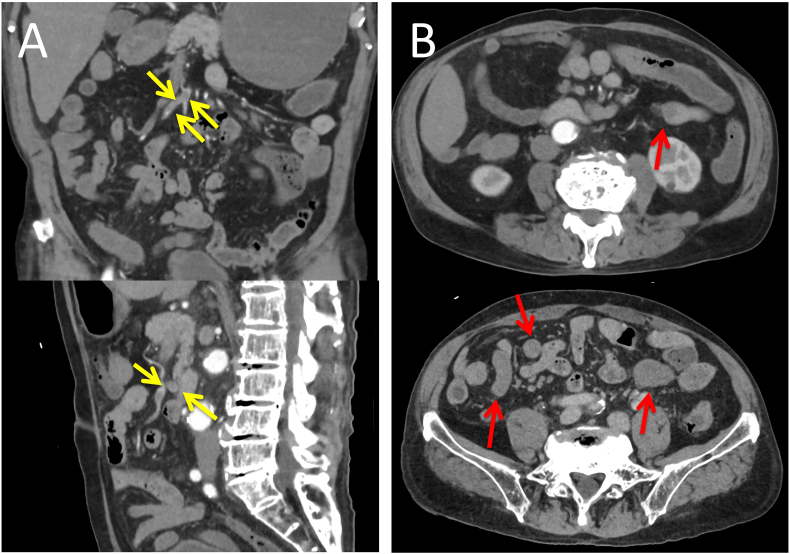
Contrasted-enhanced CT A: Occlusion of the SMA associated with a thrombus is noted. B: Contrast enhancement on the small intestine is sparse.

**Fig. 2:**
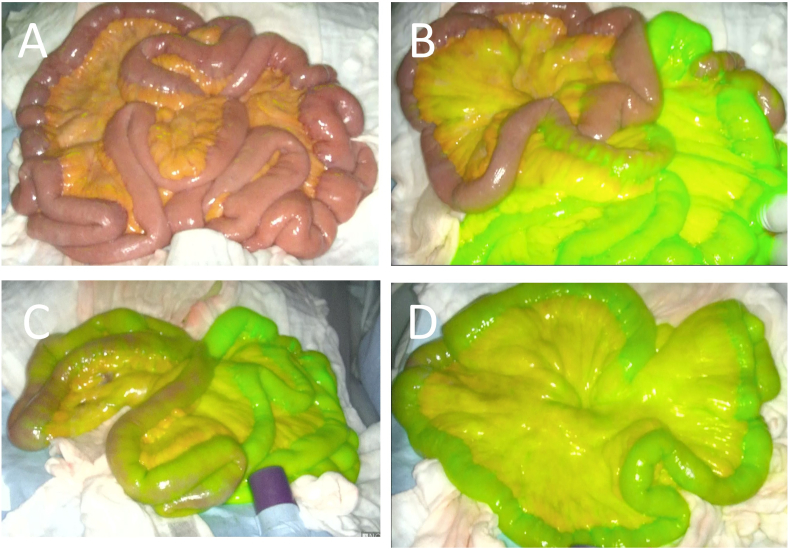
ICG Fluorescence imaging A: White light view of the small intestine. There are no signs of intestinal necrosis. B: Image three minutes after ICG injection. Emission is poor in area 20–30 cm from the ligament of Treitz, and in a large area of the ileocecal region. C: Image 3–4 min after ICG injection. The fluorescence hypointense area gradually begins to fluoresce. D: Image four minutes after ICG injection. The entire intestinal wall is showing satisfactory fluorescence emission.

**Fig. 3:**
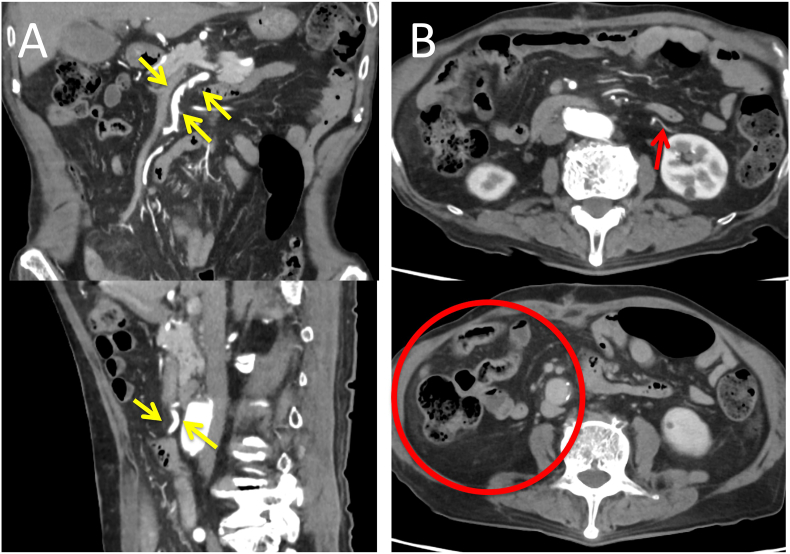
Contrast-enhanced CT on day 7 after anticoagulation A: Image showing resolution of the SMA thrombus. B: Contrast enhancement improved despite edematous changes in mesentery.

A 91-year-old man was hospitalized for acute abdominal pain and bloody bowel discharge. CT with intravenous contrast revealed superior mesenteric artery (SMA) occlusion and sparse contrast enhancement in the small intestine. Hence the patient was diagnosed with intestinal ischemia associated with SMA thrombosis (Fig. 1). Blood tests showed only mild elevations in WBC and CRP, and no decrease in Hb. There were no findings suggestive of ischemia, such as elevated lactate or CK. Cardiological examination revealed atrial fibrillation, which was presumed to be the cause of the SMA thrombosis. The patient underwent an emergency laparotomy. The operation revealed a dilated small bowel, but no macroscopic bowel necrosis was observed. To confirm adequate bowel perfusion and viability, 7.5 mg ICG was injected into peripheral blood vessels. The bowel was observed using the Stryker SPY-PHI fluorescence imaging system (Stryker Corporation). Three minutes after injection, the intestinal wall began to emit fluorescence 20–30 cm from the ligament of Treitz and in a large area of the ileocecal region. But the emission was poor. However, 4 min after the injection, the entire intestinal wall showed fluorescence (Fig. 2). The surgery was terminated without resection of the small intestine as there was no evidence of necrosis. However, there was decreased blood flow in the intestinal tract. We discussed the removal of the SMA thrombus with the cardiovascular surgeon. Due to the sparse extent of ischemia, anticoagulation therapy was decided. After 7 days of anticoagulation therapy, the abdominal symptoms were mild, and contrast-enhanced CT showed that the thrombus was resolving (Fig. 3). The patient denied any signs or symptoms of postoperative intestinal ischemia; however, he died due to dysphagic pneumonia two months after the operation.

## 3 Discussion

AMI can be classified based on abnormalities, including arterial embolism, arterial thrombosis, venous thrombosis, and nonocclusive mesenteric ischemia [5]. Acute mesenteric artery ischemia accounts for 60–70 % of all mesenteric ischemia cases, and its mortality rate has been reported to be high [1], [5]. Physicians frequently opt for exploratory laparotomy to diagnose intestinal ischemia due to AMI [2], [5]. During this procedure, physicians must assess the viability of the entire bowel, determine whether revascularization would be beneficial, and establish which sections of the intestine need to be resected. This is a difficult task with white light alone, and assessing the perfusion of the intestinal mucosa is challenging; in addition, the patterns of ischemia and necrosis vary from patient to patient [4], [5].

The evaluation of blood flow using ICG fluorescence imaging has been applied in breast reconstruction, coronary artery bypass grafting, and colorectal resection. The novel application of ICG during bowel perfusion facilitated the identification and objective assessment of the bowel ischemic zone [3]. After ICG is injected, the surgeon gains real-time visualization of which portions of the intestine receive adequate blood supply. This augmented surgical reality provided by fluorescence guidance provides the surgeon with more precise information regarding the extent of bowel necrosis and where bowel resection should be performed, if needed [5]. The imaging captures the fluorescence of indocyanine green injected into the body using a charge-coupled device camera. Indocyanine is excited by infrared light with an absorption peak at the wavelength of 780 nm and has an emission peak at 830 nm that can be easily transmitted through approximately 10 mm of human soft tissue. Intravenously injected ICG is transported to the peripheral vessels within a few seconds. In a tissue or organ where the blood flow is inhibited, the fluorescence signal is weak [2].

In the current case, the patient did not receive anticoagulation for atrial fibrillation, and the AMI was attributed to an intra-atrial thrombus. During laparotomy, the degree of necrosis of the small and large intestines was not discernible by white light visualization, until the application of ICG. ICG fluorescence showed that the blood flow to the intestinal tract was not completely interrupted. Therefore, the patient did not require an excessively invasive procedure such as resection of the small intestine. There have been few reports of fluorescent guidance being used to determine if the small intestine is damaged to the point of non-viability at the time of surgery. If this method can be utilized, as in this case, it would be a useful aid for surgeons to make an early and precise diagnosis.

## 4 Conclusion

Prompt surgical intervention of AMI is vital to save patients' lives. The uncertainty involved in diagnosis often leads to over-intervention, since surgeons must quickly determine the extent of bowel ischemia. Our case demonstrates that ICG fluorescence imaging is an effective method for determining the quality of intestinal perfusion and objective assessment of intestinal viability. This will help in the precise diagnosis and better management of AMI.

## Consent statement

Written informed consent was obtained from the patient's family for publication of this case report and accompanying images.

## Funding

None.

## Ethical approval

N/A.

## Declaration of competing interest

The authors declare that they have no conflicts of interest.
